# Feasibility study of intravascular pulsed electric field ablation for the treatment of cardiac arrhythmias

**DOI:** 10.3389/fphys.2025.1632680

**Published:** 2025-08-22

**Authors:** Zhen Wang, Yunhao Li, Ming Liang, Jingyang Sun, Jie Zhang, Lisheng Xu, Yaling Han

**Affiliations:** ^1^ Department of Cardiology, General Hospital of Northern Theater Command, Shenyang, China; ^2^ College of Medicine and Biological Information Engineering, Northeastern University, Shenyang, China; ^3^ National Key Laboratory of Frigid Zone Cardiovascular Diseases, General Hospital of Northern Theater Command, Shenyang, China; ^4^ College of Information Science and Engineering, Northeastern University, Shenyang, China

**Keywords:** pulsed electric field ablation technique, arrhythmia, endovascular ablation, computer simulation, electric field prediction, temperature assessment

## Abstract

**Background:**

Pulsed electric field ablation (PFA) techniques for treating cardiac arrhythmias have attracted considerable interest. For example, atrial fibrillation can be effectively treated by pulmonary vein isolation using PFA. However, some arrhythmias originate deep within the myocardium, making them difficult to reach with conventional ablation methods. Therefore, this study aimed to explore endovascular catheter-based ablation using computational modeling to assess the electric field and temperature distributions during the procedure.

**Methods:**

A three-dimensional computer model of the ablation catheter and heart was developed. The catheter was positioned within the heart model to simulate endovascular ablation, and the ablation damage range was estimated using the 1000 V/cm contour. Additionally, a probe function was used to monitor the maximum electric field and temperature within the ablation zone to evaluate the feasibility and safety of this approach.

**Results:**

The electric field can penetrate blood vessels and fat to induce effective myocardial injury. The extent of myocardial damage increases with higher pulse voltages; however, excessive voltage may also damage blood vessels (vascular damage threshold: 3500 V/cm). An appropriate electrode configuration can achieve a more uniform myocardial injury across different cross-sections. Temperature rise near the catheter electrode is significant, but appropriate pulse interval settings can prevent thermal damage in the target area (simulated maximum temperature: 46.8 °C; thermal damage threshold for biological tissue: 55 °C).

**Conclusion:**

Intravascular pulsed electric field ablation can effectively damage the myocardium without harming blood vessels when suitable pulse parameters are applied. The ablation device settings strongly influence the maximum temperature in the ablation zone and help limit thermal effects. These findings support the feasibility of using small endovascular catheters to treat cardiac arrhythmias.

## 1 Introduction

Arrhythmia is an irregular heartbeat caused by abnormal cardiac electrophysiologic activity. It carries a risk of heart failure and stroke, and severe arrhythmias may result in patient death (Arevalo et al., 2016; Xie et al., 2022; Curtain et al., 2021; Arbelo et al., 2023). In recent years, pulsed electric field ablation (PFA) has been explored as a treatment for arrhythmias. This technique stimulates cells and creates small pores in cell membranes, leading to intracellular homeostasis disruption and cell death. PFA has been widely applied to treat atrial fibrillation by isolating the pulmonary veins (Koruth et al., 2019; Cochet et al., 2021; Meckes et al., 2022). Stewart et al. demonstrated the efficacy of PFA in an animal study using a circular ablation catheter to target the endocardium. This trial showed that PFA had a better safety profile than radiofrequency ablation due to the absence of inflammation and arterial damage. Other researchers performed pulmonary vein isolation in patients with atrial fibrillation. Postoperative examination showed persistent transmural cardiomyopathy without side effects such as pulmonary vein stenosis or mucosal lesions. This study demonstrated the safety and efficacy of PFA for isolating the pulmonary veins and the posterior wall of the left atrium (Stewart et al., 2019; Reddy et al., 2020). In an animal study of epicardial ablation, the catheter effectively damaged myocardial tissue without complications. No injury to adjacent tissues (e.g., esophagus, pulmonary veins) was observed, demonstrating the safety and feasibility of epicardial PFA. A three-dimensional computational modeling study found that the results of the simplified and complex models were nearly identical, reducing the computational burden of simulations (Padmanabhan et al., 2019; González-Suárez et al., 2022a). Evaluation of epicardial PFA using three-dimensional modeling revealed that the presence of nerves within fat layers may distort electric field distribution due to the higher conductivity of neural tissue. In a subsequent study on intracoronary metallic stents, it was found that metal stents in the ablation region distorted the electric field and increased tissue temperature. However, this temperature change did not cause thermal damage to the target area (Scheffer et al., 2016; González-Suárez et al., 2022b).

Previous studies have demonstrated the effectiveness of PFA in treating arrhythmias. Target sites include the pulmonary veins for atrial fibrillation and the ventricular wall for ventricular tachycardia. However, some arrhythmias originate from deep myocardial structures that are challenging to reach with conventional techniques, making epicardial ablation particularly difficult. Therefore, we propose using a small catheter-based needle to perform ablation within cardiac vessels to treat these arrhythmias. This approach leverages the different electric field thresholds of various cardiac tissues (Kaminska et al., 2012; Ramirez et al., 2020). We developed computational models of the ablation catheter and target area and assessed the ablation region using electric field and bioheat transfer modules. Simulations were conducted to evaluate the feasibility of endovascular ablation for treating arrhythmias. Temperature was also assessed to account for the heating effects of PFA in the target region. To our knowledge, this is the first computational simulation study on endovascular ablation for the treatment of cardiac arrhythmias.

## 2 Methods

### 2.1 Model building

**FIGURE 1:**
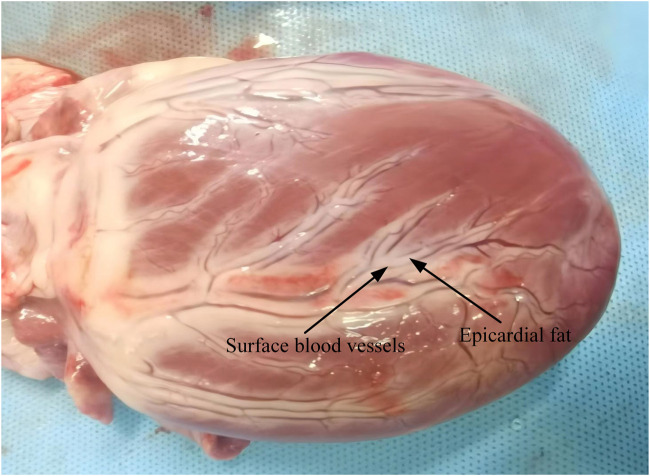
Schematic diagram of blood vessels on the external surface of the heart (Xuan et al., 2025).

**FIGURE 2:**
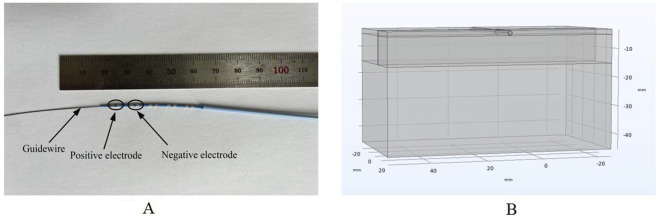
Computer simulation modeling (Meckes et al., 2022; González-Suárez et al., 2022b; Ramirez et al., 2020), (The ablation electrodes are distributed as shown in Panel **(A)**. During the simulation calculation, two adjacent electrodes are set as positive electrodes, and the other two adjacent electrodes are set as negative electrodes. There are no other ablation electrodes between the positive and negative electrodes). **(A)** Ablation catheters. **(B)** Three-dimensional computational models.

To effectively analyze the electric field and heat distribution in endovascular ablation, we simplified the real heart model and constructed a computational model. Our computational model employed the actual dimensions of the ablation catheter and applied a layered approach to cardiac tissues (González-Suárez et al., 2022a; Sano et al., 2017). Based on the distribution of cardiac tissues and previous analytical models, we segmented the ablation target region into the fat, myocardial, and blood layers. The fat layer contains blood vessels, and the ablation catheter is positioned centrally within them. This model simplification is based on the actual structural characteristics of the tissue (González-Suárez et al., 2022a; González-Suárez et al., 2022b). A schematic representation of cardiac blood vessel distribution is provided in Figure 1 (González-Suárez et al., 2022b; Song et al., 2021; Habib et al., 2009). In the computational model, the ablation catheter was equipped with multiple discharge electrodes. Each pair of electrodes served as a positive discharge unit, with a 2 mm spacing to simulate the 3F pulse ablation catheter parameters. The ablation catheter was set to have a fat layer thickness of 1.8–1.9 mm and a myocardial layer thickness of 10 mm, and the length and width of the model were set to 80 mm and 40 mm, respectively, concerning previous studies (González-Suárez et al., 2022b; González-Suárez et al., 2022c; Caluori et al., 2020). The computer simulation model is shown in Figure 2. In Figure 2B, only the regions of biological tissue potentially affected by PFA were considered, as these tissues play a key role in the development and maintenance of arrhythmias. This simplified approach has been validated as feasible in previous simulation studies of pulsed ablation injury (Meckes et al., 2022; González-Suárez et al., 2022a; González-Suárez et al., 2022b).

### 2.2 Boundary conditions and pulse parameters

In the model’s pulse parameter settings, the ablation catheter metal electrode was set to 800–1,000 V, with a pulse duration of 100 µs. No specific pulse interval was defined, as this simulation is an *a priori* study (González-Suárez et al., 2022b; Al-Khadra et al., 2000). The middle insulator of the ablation metal electrode and the model surface were set to 0 V to ensure that pulse energy was confined within the computational model. Additionally, the ablation catheter metal electrode was configured with both positive and negative poles (Padmanabhan et al., 2019; Sano et al., 2010).

In catheter-based endovascular ablation, the ablation catheter directly contacts the blood vessel. During energy release from the catheter electrodes, the targeted ablation area experiences a temperature rise due to pulsed energy. Previous studies have shown that the temperature threshold for thermal damage in biological tissues is approximately 55 °C (Wood et al., 2011; Zang et al., 2024). To determine whether the ablation region reaches a temperature that causes tissue damage and to accurately assess temperature changes, we incorporated a biological heat transfer module into the computational model (González-Suárez et al., 2022b; Zang et al., 2024). The thermal boundary conditions of the computational model included a set temperature of 37 °C and a thermal convection coefficient of 1,417 W/m^2^K at the myocardium-blood boundary, corresponding to a myocardial blood flow rate of 24.4 cm/s. In the cardiac vasculature, we simplified blood flow by assuming a velocity of 0.5 m/s, corresponding to a thermal convection coefficient of 63.19 W/m^2^K (Anjaneyulu et al., 2008; Tungjitkusolmun et al., 2001; González-Suárez and Berjano, 2015).

### 2.3 Material properties

Variations in the conductivity of biological tissues significantly impact ablation calculations. Assigning appropriate conductivity values in the model enhances the accuracy of damage assessment in the ablation region. Studies have shown that the conductivity of biological tissues changes upon electrical stimulation. Before exposure to a pulsed electric field, the biological membrane remains intact, resulting in low conductivity. However, after receiving pulse energy, the electric field induces the formation of microscopic pores in the cell membrane, increasing tissue conductivity. Over time, the overall conductivity of biological tissues follows an S-shaped curve (González-Suárez et al., 2022c; Avazzadeh et al., 2022; Howard et al., 2022). The effect of bio-thermal changes on tissue conductivity was incorporated into the computational simulation, where conductivity increased by 2% with rising temperature. In the computational simulation, a sigmoid function was used to model conductivity changes in various cardiac tissues (myocardium, fat, blood vessels, etc.). The parameters used in the electric field simulation are summarized in Table 1 (González-Suárez et al., 2022b; Lei et al., 2021; Zang et al., 2020; Ayuttaya and Rattanadecho, 2021; Wang et al., 2023).

$$\sigma \left(E,T\right)=\left(\sigma_{0}+\frac{\sigma_{1}-\sigma_{0}}{1+10e^{-}\frac{\left(\left|E\right|-58,000\right)}{3000}}\right)\cdot 1.02^{T-37}$$

**TABLE 1:** Parameters of model electrical calculations.

Mlement	Electrode	Poyurethane	Myocardium	Vessel	Blood	Fat
σ _0_(S/m)	4.6e^6^	1e^−5^	0.0537	0.4	0.7	0.0377
σ _1_(S/m)			0.281	0.67	0.748	0.0438

In the table, σ represents conductivity. The conductivity of non-biological tissue remains unchanged during ablation by default.

In the formula, the conductivity of biological tissues before and after electroporation, represented by σ _0_ and σ _1_ respectively, is provided in Table 1. The difference arises primarily because the ablation process induces pore formation in the cell membrane, which subsequently increases conductivity. Non-biological tissues exhibited no change in conductivity values in the simulations.

### 2.4 Control equations

The computational model involves electro-thermal coupling. For computational software, we selected COMSOL due to its proven effectiveness in simulating electro-thermal interactions in the ablation region. In this study, we utilized the software’s capabilities for 3D model construction and appropriate meshing. The ablation catheter was incorporated into the cardiac vessels to simulate real human vascular ablation (González-Suárez et al., 2022b; González-Suárez et al., 2022c; Zang et al., 2024). The computational model incorporates Laplace’s equation to simulate the ablation region.

$$\nabla \cdot \left(\sigma \nabla V\right)=0$$

E=−∇V

J=σE

Where σ is the conductivity (S/m), the values used in the calculation are shown in the table above; E is the electric field strength (V/cm); V is the voltage (V); J is the current density (A/m^2^).

For the thermal response induced by the ablation region, we introduced the bioheat equation into the computational model to account for the thermal damage that the ablation catheter may cause to the targeted ablation region. In the computational modeling, we computationally simulate the heat generated in the ablation region through the ablation time.

$$Q=\sigma {\left|E\right|}^{2}$$

$$\rho c_{p}\frac{dT}{dt}=\nabla \cdot \left(k\nabla T\right)+Q+Q_{e}+Q_{m}$$

Where ρ is the tissue density (kg/m^3^); c is the specific heat (J/kg·K); T is the temperature (°C); t is the time (s); k is the thermal conductivity (W/m·K); Q is the heat generated by the electric field (W/m^3^), Q_e_ is the heat loss due to the blood flow (W/m^3^), and Q_m_ is the heat loss due to the bio-metabolism (W/m^3^), and the magnitude of all three is related to the field strength and the conductivity. The calculation parameters used for the calculation of the thermal response in the ablation region are shown in Table 2 (González-Suárez et al., 2022b; Lei et al., 2021; Zang et al., 2020; Ayuttaya and Rattanadecho, 2021; Wang et al., 2023).

**TABLE 2:** Calculated parameters for the bioheat control equation.

Mlement	Electrode	Poyurethane	Myocardium	Vessel	Blood	Fat
k(W/m·K)	71	23	0.56	0.5	0.52	0.21
ρ (kg/m^3^)	21500	1440	1081	1100	1050	911
c(J/kg·K)	132	1050	3686	3400	3617	2348

In the table, k is the thermal conductivity, ρ is the density, and c is the specific heat.

### 2.5 Analysis of results

In this study, we used finite element analysis software to evaluate the ablation damage range in cardiac vascular catheter ablation. Additionally, considering the close proximity of the ablation catheter to the cardiac vasculature and the thermal effects of pulse energy, we assessed the temperature distribution in the ablation region during the simulation. We simulated different scenarios, including variations in cardiac vessel diameter and fat layer thickness. To assess the effective ablation damage range, we used the 1000 V/cm contour as the criterion for vascular ablation. Previous studies indicate that under identical pulse conditions, PFA induces myocardial damage at 1000 V/cm, while vascular endothelium damage occurs at electric field strengths of up to 3500 V/cm (Ramirez et al., 2020; Avazzadeh et al., 2021; Maor et al., 2009). The varying damage thresholds of different tissues to pulsed electric fields enable catheter-based endovascular ablation. Following ablation catheter discharge, we simulated temperature recovery in the ablated area, aiding in parameter optimization for future clinical studies.

## 3 Results

### 3.1 Electric field distribution

**FIGURE 3:**
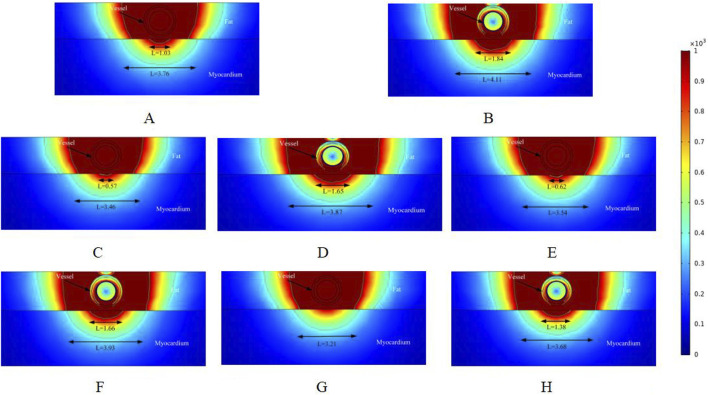
Electric field distribution in the ablation region of the PFA at 800 V electrode voltage (Starting from the upper left corner, odd numbers show the ablation effect between the two ablation electrodes of the catheter, and even numbers show the ablation effect in the center of the catheter’s ablation electrodes). **(A)** Fat 1.8 mm, vessel 0.8 mm. **(B)** Fat 1.8 mm, vessel 0.8 mm. **(C)** Fat 1.8 mm, vessel 0.75 mm. **(D)** Fat 1.8 mm, vessel 0.75 mm. **(E)** Fat 1.9 mm, vessel 0.8 mm. **(F)** Fat 1.9 mm, vessel 0.8 mm. **(G)** Fat 1.9 mm, vessel 0.75 mm. **(H)** Fat 1.9 mm, vessel 0.75 mm.

**FIGURE 4:**
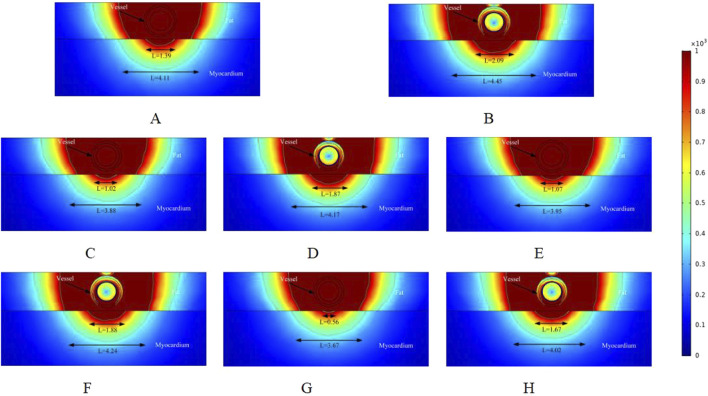
Electric field distribution in the ablation region of the PFA at 800 V electrode voltage (Starting from the upper left corner, odd numbers show the ablation effect between the two ablation electrodes of the catheter, and even numbers show the ablation effect in the center of the catheter’s ablation electrodes). **(A)** Fat 1.8 mm, vessel 0.8 mm. **(B)** Fat 1.8 mm, vessel 0.8 mm. **(C)** Fat 1.8 mm, vessel 0.75 mm. **(D)** Fat 1.8 mm, vessel 0.75 mm. **(E)** Fat 1.9 mm, vessel 0.8 mm. **(F)** Fat 1.9 mm, vessel 0.8 mm. **(G)** Fat 1.9 mm, vessel 0.75 mm. **(H)** Fat 1.9 mm, vessel 0.75 mm.

**FIGURE 5:**
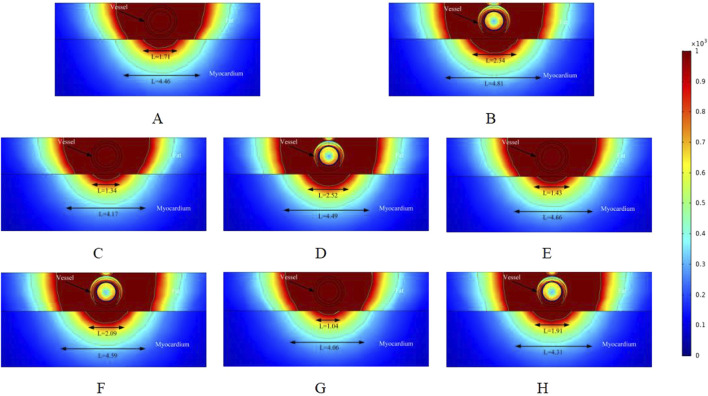
Electric field distribution in the ablation region of the PFA at 800 V electrode voltage (Starting from the upper left corner, odd numbers show the ablation effect between the two ablation electrodes of the catheter, and even numbers show the ablation effect in the center of the catheter’s ablation electrodes). **(A)** Fat 1.8 mm, vessel 0.8 mm. **(B)** Fat 1.8 mm, vessel 0.8 mm. **(C)** Fat 1.8 mm, vessel 0.75 mm. **(D)** Fat 1.8 mm, vessel 0.75 mm. **(E)** Fat 1.9 mm, vessel 0.8 mm. **(F)** Fat 1.9 mm, vessel 0.8 mm. **(G)** Fat 1.9 mm, vessel 0.75 mm. **(H)** Fat 1.9 mm, vessel 0.75 mm.

Figures 3–5 illustrate the electric field distribution in the targeted ablation region under different pulse voltages. Specifically, Figure 3 presents the effects of varying fat thickness and vessel diameter on the electric field distribution. The targeted area was ablated using an electrode release voltage of 800 V and a pulse duration of 100 µs. To simplify the intravascular electric field ablation scenario, we simulated only the case where the ablation catheter was positioned at the vessel center. In this simulation, we analyzed only the cross-section of the ablation result, as the axial cross-section of the effective injury area can be controlled by adjusting the catheter’s position.

As shown in Figures 3A,B illustrate the electric field distribution when the fat thickness of the target area is 1.8 mm and the blood vessel radius is 0.8 mm. The results indicate that part of the myocardium is effectively damaged (electric field >1000 V/cm), with effective ablation widths of 1.03 mm and 1.84 mm, respectively. Additionally, by setting the electric field strength threshold at 400 V/cm, we identified another set of effective ablation regions, with widths of 3.76 mm and 4.11 mm, respectively. This phenomenon occurs because, as reported in previous studies, variations in pulse width alter the myocardium’s sensitivity to electric field strength when the electrode release voltage remains constant (González-Suárez et al., 2022a; Kaminska et al., 2012). When all other parameters remained unchanged and the vessel radius decreased to 0.75 mm, the effective ablation area of the pulsed electric field on the myocardium was altered. The effective myocardial injury widths in Figures C and D were 0.57 mm and 1.65 mm, respectively. Additionally, when using an electric field strength of 400 V/cm as the injury threshold, the ablation injury widths (3.46 mm and 3.87 mm) were smaller than those in Figures A and B.

Figures E and F illustrate the ablation effect in the ablation region with a fat thickness of 1.9 mm, in comparison to Figures A and B, where the fat thickness parameter was varied. For Figures E and F, the effective myocardial injury widths were 0.62 mm and 1.66 mm, respectively, with ablation injury widths of 3.54 mm and 3.93 mm when the electric field strength was set to 400 V/cm. The results are summarized below. When the fat thickness was 1.9 mm and the blood vessel radius was 0.75 mm, Figure G showed no effective damage to the myocardium, while Figure H showed a myocardial injury width of 1.38 mm (3.32 mm versus 3.68 mm when the electric field strength was set to 400 V/cm). This suggests that fat thickness and vessel diameter may significantly influence the effective ablation injury width when the pulse voltage remains constant. Note that in Figure 3, the vessels were subjected to an electric field strength greater than 1000 V/cm. Based on the above, it is clear that the electric field threshold for inducing effective damage to the vessels in this simulation was 3500 V/cm. The specific electric field values experienced by the vessels in the various computational simulations will be provided in Section 3.3 below.

In the simulation, we adjusted the pulse voltage of the ablation catheter, and Figure 4 illustrates the ablation effect on the targeted tissue region when the pulse voltage was set to 900 V. Figure 4 shows that the ablation electric field caused effective damage to the myocardium. Compared to Figure 3, the ablation damage range gradually expanded, indicating that electric field intensity plays a significant role in the effective damage range of the ablation region. As shown in Figure 3, we analyzed only the cross-section of the ablation catheter. In Figures 4A,B, the effective myocardial injury widths were 1.39 mm and 2.09 mm (4.11 mm and 4.45 mm when the electric field strength was set to 400 V/cm). Compared to the corresponding locations in Figure 3, the myocardial effective ablation injury width increased. In Figure 3, the myocardial effective injury ranges were 0 mm and 1.38 mm in Figures E and F, and 0.56 mm and 1.67 mm in Figure 4. This indicates that within the safe electric field strength range, the effective injury range of the ablation region can be increased by adjusting the voltage strength of the ablation catheter electrodes. Changing the electric field threshold (400 V/cm) for the ablation region similarly altered the effective damage range of the myocardium, which could be further adjusted by varying the intensity of the ablation catheter release voltage.

To better understand how changes in the ablation catheter release voltage affect the effective damage range of the myocardium, we adjusted the ablation catheter voltage to 1000 V in the simulation. The ablation results are shown in Figure 5. Comparing Figures 3, 4, we can observe that the effective ablation electric field strength (greater than 1000 V/cm) penetrates the fat layer and causes damage to the myocardial layer. Figure 5G shows that the effective myocardial damage ranges were 1.04 mm and 4.06 mm. This damage range was the largest observed for different voltage settings of the ablation catheter. Additionally, the effective myocardial ablation damage range increased compared to the corresponding ablation region cross-sections in Figures 3, 4. From Figures 3–5, we can observe that the effective ablation injury range varies significantly at different cross-sections of the ablation catheter. The myocardial injury range was smaller in odd-numbered cross-sections than in even-numbered ones (with ablation parameters remaining unchanged and only the cross-sections varying). The effective damage range of ablation was smaller in the odd-numbered cross-sections than in the even-numbered ones.

### 3.2 Temperature distribution

This study is the first computational simulation of intravascular ablation for arrhythmia treatment, with a focus on the temperature rise in the targeted ablation region caused by pulsed electric field energy. To date, no validated assessment of ablation temperature for vascular ablation exists, and the temperature rise in the ablation region may cause thermal damage (González-Suárez et al., 2022b; Haines, 2011). In this study, we assessed the ablation region temperature from the same cross-section as the electric field distribution, which helps identify the type of energy causing effective damage to the biological tissue.

**FIGURE 6:**
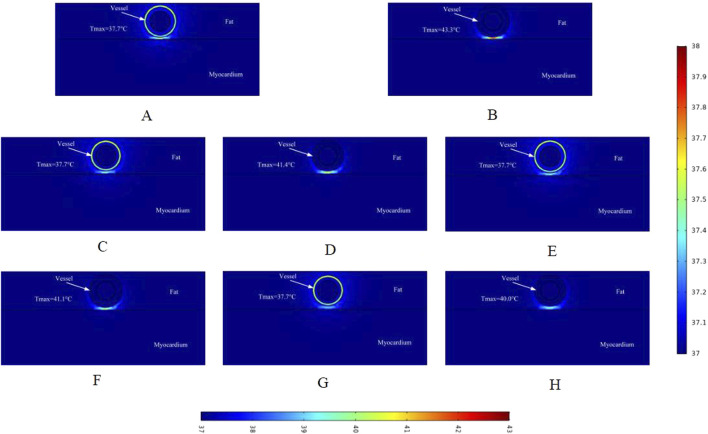
Temperature distribution in the ablation region of the PFA at 800 V electrode voltage (Starting from the upper left corner, odd numbers show the ablation effect between the two ablation electrodes of the catheter, and even numbers show the ablation effect in the center of the catheter’s ablation electrodes). Refer to the legend on the lower side of the figure for the center ablation effect of the catheter ablation electrode. **(A)** Fat 1.8 mm, vessel 0.8 mm. **(B)** Fat 1.8 mm, vessel 0.8 mm. **(C)** Fat 1.8 mm, vessel 0.75 mm. **(D)** Fat 1.8 mm, vessel 0.75 mm. **(E)** Fat 1.9 mm, vessel 0.8 mm. **(F)** Fat 1.9 mm, vessel 0.8 mm. **(G)** Fat 1.9 mm, vessel 0.75 mm. **(H)** Fat 1.9 mm, vessel 0.75 mm.

As shown in Figure 6, in the cross-section without ablation electrodes, the tissue temperature in the ablation region was 37.7 °C. (Note that we assessed the temperature to one decimal place, and the temperature variation between ablation regions was minimal.) This indicates that in the cross-section without ablation electrodes, the temperature in the ablation region remains relatively stable. Figures B and D show that the highest temperatures in the ablation regions were 43.3 °C and 41.4 °C, respectively. In these two regions, we only changed the vessel diameter in the model, suggesting that vessel diameter affects the temperature of the ablation region. Comparing Figure D and Figure F, with other parameters unchanged and only the fat thickness changed, the tissue temperature in the ablation region decreased by 0.3 °C, indicating that fat thickness hinders the increase in tissue temperature. Comparing Figures F and H, the tissue temperature in the ablation region decreased by 1.1 °C with changes in vessel diameter, consistent with the previous results.

**FIGURE 7:**
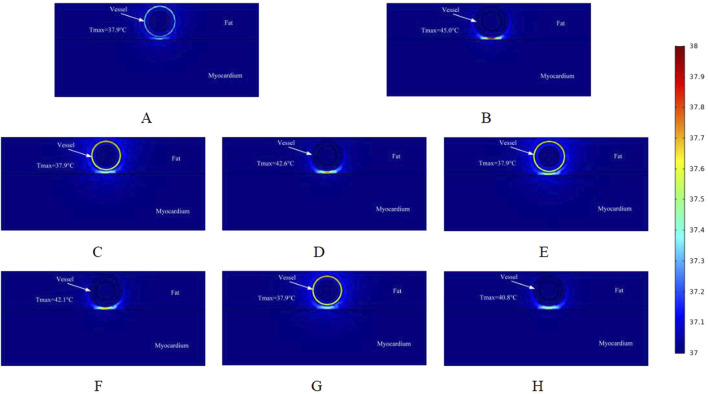
Temperature distribution in the ablation region of the PFA at 900 V electrode voltage (Starting from the upper left corner, odd numbers show the ablation effect between the two ablation electrodes of the catheter, and even numbers show the ablation effect in the center of the catheter’s ablation electrodes). **(A)** Fat 1.8 mm, vessel 0.8 mm. **(B)** Fat 1.8 mm, vessel 0.8 mm. **(C)** Fat 1.8 mm, vessel 0.75 mm. **(D)** Fat 1.8 mm, vessel 0.75 mm. **(E)** Fat 1.9 mm, vessel 0.8 mm. **(F)** Fat 1.9 mm, vessel 0.8 mm. **(G)** Fat 1.9 mm, vessel 0.75 mm. **(H)** Fat 1.9 mm, vessel 0.75 mm.

Similar to the electric field distribution study, we made a simulation of the temperature effects caused by different voltages on the ablation region, and Figure 7 shows the schematic distribution of the maximum temperatures in different cross-sections of the targeted ablation region at an ablation catheter pulse voltage of 900 V. The maximum temperatures of the ablated tissues in different cross-sections of the ablation catheter are shown in Figure 7. Comparing with Figure 6, it can be seen that in the cross-section without ablation electrodes, the maximum temperature of the ablated tissue obtained from the computational simulation increased by 0.2 °C (e.g., 37.7 °C in Figure 6A, 37.9 °C in Figure 7A). Meanwhile, within this cross-section, fat thickness and vessel diameter had less effect on the rise in tissue temperature in the ablation region. Figure 7B shows that the maximum temperature in the ablation region was 45.0 °C, the fat thickness was 1.8 mm and the vessel radius was 0.8 mm in this computational model. At the same time, the maximum temperatures in different cross-sections of the ablation region increased compared with Figure 6, and the maximum temperature in the same cross-section increased by 1.7 °C (e.g., 43.3 °C in Figure 6B, 45.0 °C in Figure 7B). The pulse voltage released by the ablation catheter has a positive effect on the elevation of tissue temperature in the ablation region.

**FIGURE 8:**
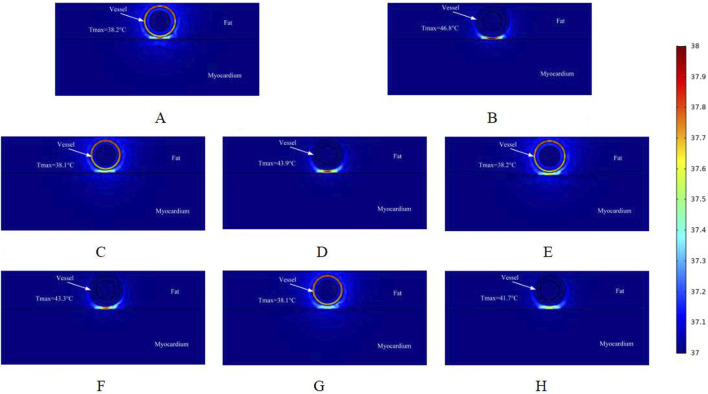
Temperature distribution in the ablation region of the PFA at 1000 V electrode voltage (Starting from the upper left corner, odd numbers show the ablation effect between the two ablation electrodes of the catheter, and even numbers show the ablation effect in the center of the catheter’s ablation electrodes). **(A)** Fat 1.8 mm, vessel 0.8 mm. **(B)** Fat 1.8 mm, vessel 0.8 mm. **(C)** Fat 1.8 mm, vessel 0.75 mm. **(D)** Fat 1.8 mm, vessel 0.75 mm. **(E)** Fat 1.9 mm, vessel 0.8 mm. **(F)** Fat 1.9 mm, vessel 0.8 mm. **(G)** Fat 1.9 mm, vessel 0.75 mm. **(H)** Fat 1.9 mm, vessel 0.75 mm.

Figure 8 shows the effect of the maximum temperature distribution in the ablation region at a pulse voltage of 1000 V. We also monitored the temperatures of different cross-sections, and compared with the pulse voltage of 800 V and 900 V, the pulsed electric field generates more heat to the targeted ablation region, and thus the maximum temperatures shown in different cross-sections of the ablation region are larger than the former two.

As shown in Figure 8B, the highest temperature in the ablation region in this simulation is 46.8 °C, which is an increase of 1.8 °C compared with the pulse voltage of 900 V. Compared with the region without ablation electrodes, the highest temperature in the ablation cross-section was 38.2 °C. In the sub-section, different fat layer thicknesses and different vessel radii had a small effect on the temperature of the ablation region (only 0.1 °C). Comparing Figures 8B,H, there was a temperature difference of 5.1 °C at the same voltage. When ablation electrodes were present in the ablation region, different fat layer thicknesses and different vessel radii had a greater effect on the temperature of the ablation region.

### 3.3 Electric field and temperature (maximum) monitoring

**FIGURE 9:**
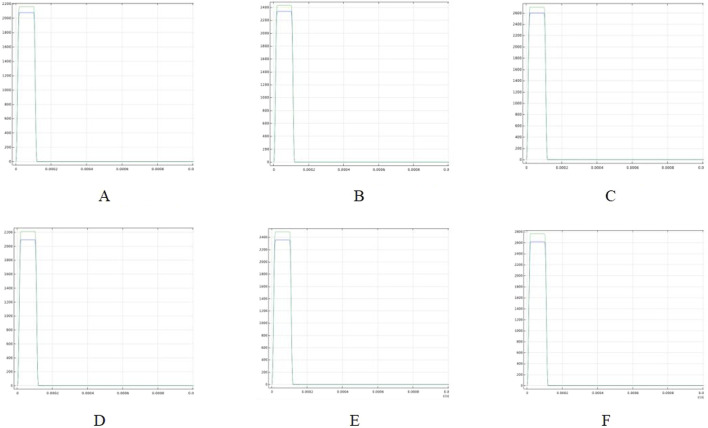
Highest electric field thresholds in the ablation region for different voltages (Panels **(A–C)** show the maximum electric field in the ablation region for a fat thickness of 1.8 mm and a vessel radius of 0.8 mm; Panels **(D–F)** show the maximum electric field in the ablation region for a fat thickness of 1.9 mm and a vessel radius of 0.75 mm). **(A)** Voltage 800 V. **(B)** Voltage 900 V. **(C)** Voltage 1000 V. **(D)** Voltage 800 V. **(E)** Voltage 900 V. **(F)** Voltage 1000 V.

Cardiac blood vessels supply oxygen and nutrients to the myocardium and facilitate the removal of metabolic waste. Endovascular ablation is performed to target the myocardium with electric field energy, which penetrates the blood vessels and surrounding tissues. Therefore, it is crucial to investigate the maximum electric field strength applied to the cardiac vessels during ablation. Figure 9 illustrates the maximum electric field strength applied to blood vessels during ablation at different catheter voltages. Figure C shows that with a fat thickness of 1.8 mm and a vessel radius of 0.8 mm, the blood vessel tissues were exposed to an electric field strength of 2700 V/cm at an ablation voltage of 1000 V. This is the maximum electric field strength in the simulation, which is lower than the threshold of 3500 V/cm required to effectively damage the myocardium (Wang et al., 2023).

This suggests that the vascular tissue is not damaged by the electric field under these conditions. Additionally, as the ablation catheter’s pulse voltage decreased, the maximum electric field strength to which the vessels were exposed decreased to 2160 V/cm. Figures D through F show the maximum electric field strength applied to the vessels under different parameters (fat thickness of 1.9 mm and vessel radius of 0.75 mm). As shown in Figure F, the maximum electric field strength applied to the vessels was 2765 V/cm at a catheter release voltage of 1000 V. The maximum electric field strengths for both parameters indicate that the vascular tissue was unaffected by the electric field, even when the myocardium experienced effective electric field injury.

**FIGURE 10:**
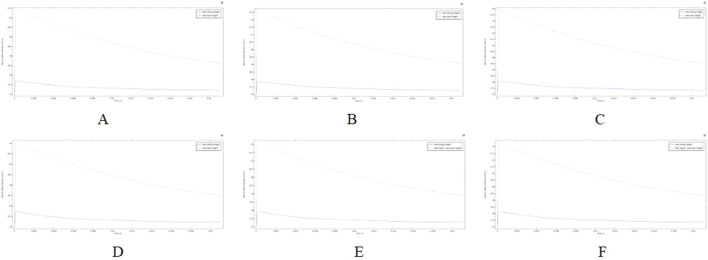
Temperature variation with time in the ablation region for different voltages (Panels **(A–C)** show temperature drop curves in the ablation region for a fat thickness of 1.8 mm and a vessel radius of 0.75 mm; Panels **(D–F)** show temperature drop curves in the ablation region for a fat thickness of 1.9 mm and a vessel radius of 0.8 mm). **(A)** Voltage 800 V. **(B)** Voltage 900 V. **(C)** Voltage 1000 V. **(D)** Voltage 800 V. **(E)** Voltage 900 V. **(F)** Voltage 1000 V.

Excessive temperature in the ablation region can cause thermal damage to surrounding tissues, making the control of ablation catheter pulse parameters an important aspect of the study. In this study, we adjusted the pulse interval of the ablation catheter’s release voltage and obtained the temperature decay curve over time in the targeted ablation region. Figure 10 illustrates the temperature profiles for two computational models. It shows that when the ablation electrode was present in the region (fat thickness of 1.8 mm and vessel radius of 0.78 mm), the temperature in the ablation region (42.6 °C) was significantly higher than in the other cross-section (37.9 °C) as shown in Figure B. The temperature in the ablation region decreased initially at a faster rate and then more slowly, reaching 40.4 °C at 0.01 s and 39.2 °C at 0.02 s. Although we did not simulate when the temperature dropped to 37 °C, the data suggest that the pulse interval is a crucial parameter to consider during PFA, as it significantly affects the temperature of the ablation region.

## 4 Discussion

### 4.1 Findings

Pulsed electric field ablation (PFA), an emerging treatment for arrhythmia, has garnered significant attention from clinical researchers in recent years. It destroys cardiomyocytes by applying high-intensity pulsed energy to myocardial tissues, while causing less damage to surrounding tissues (Koruth et al., 2019; Napotnik et al., 2021). Numerous studies have shown that using ablation catheters to isolate the pulmonary veins can effectively treat atrial fibrillation. This is because ablation energy can penetrate pulmonary vein tissue, causing effective damage to the surrounding myocardial tissue (Reddy et al., 2020; Jiang et al., 2024; Sugrue et al., 2022). Studies have also shown that electric field ablation of the targeted area through the epicardium can effectively damage the myocardium. Additionally, some researchers have used computer simulations of epicardial ablation to analyze the electric field and heat distribution in the targeted ablation area (González-Suárez et al., 2022b; Wang et al., 2024a; Madhavan et al., 2016).

However, up to now, no study has been conducted to achieve the treatment of arrhythmia by PFA from the cardiac vasculature, and this simulation study provides a new idea for clinical researchers to reach the deep myocardium from the vascular route to treat arrhythmia. To our knowledge, this is the first computational simulation study to simulate both the electric field and thermal distribution in the targeted ablation area during intravascular ablation in the heart. In this study, we used computational modeling and finite element simulations to assess the ablation area, considering changes in parameters such as fat thickness and blood vessel radius. The main findings of this study are as follows:

1. Pulsed electric field ablation in the cardiac vasculature for the treatment of arrhythmias is feasible because, with appropriate pulse parameters, the maximum value of the electric field in the ablation area is less than the threshold for vascular injury and greater than the threshold for effective myocardial injury (the threshold for myocardial injury is 1,000 V/cm, and the threshold for vascular injury is 3,500 V/cm).
2. When the pulse voltage is unchanged, the effective myocardial injury range of the targeted ablation area changes when the fat thickness and the radius of the blood vessel change, and the thickening of the fat thickness and the smaller radius of the blood vessel have a certain impediment to the strength of the electric field, which will cause the effective myocardial injury range to become smaller. The pulse voltage released by the catheter has a positive effect on the ablation area, and increasing the pulse voltage increases the effective myocardial injury range when other parameters are held constant. However, it is worth noting that too much pulse voltage may cause damage to the vasculature.
3. At different cross-sections of the ablation catheter, the effective ablation damage range of the pulsed electric field to the myocardium is changing, which may cause the damage to the targeted ablation area to fail to meet the target requirements, and reasonable catheter ablation electrode pairing may reduce this range of variation. When ablation electrodes are present in the cross-section of the ablation catheter, the temperature of the targeted ablation region is higher than the temperature of the other cross-section, with similar conclusions for different pulse voltages, while the temperature variation in this cross-section is strongly influenced by the fat thickness and the radius of the vessel (as shown in Figures 6–8).
4. During cardiac endovascular ablation, the temperature of the tissue in the ablation area increases dramatically, especially in the vicinity of the ablation electrode. Therefore, after an ablation pulse, it is recommended to wait for a short period for the temperature in the ablation area to decrease to a normal level before the next pulse is released.

Numerous clinical studies have demonstrated the distinct safety advantages of PFA, due to the differential sensitivity of biological tissues to electric fields (Ramirez et al., 2020; Reddy et al., 2019). The effectiveness of PFA in achieving pulmonary vein isolation for the treatment of atrial fibrillation has been well validated. Studies have also demonstrated the applicability of PFA for the ablation of supraventricular and ventricular tachycardia (Xie et al., 2022; Arbelo et al., 2023; Reddy et al., 2020). However, certain arrhythmias originate deep within the myocardium, making it challenging for conventional ablation catheters to access the target region. Therefore, our simulation and modeling studies on cardiac venous ablation may provide valuable clinical insights into this therapeutic strategy.

Ablation parameters are key determinants of tissue injury during ablation. Variations in ablation parameters can alter myocardial sensitivity to electric field intensity, thereby shifting the damage threshold of surrounding vasculature (Kaminska et al., 2012; Maor et al., 2009). Therefore, optimizing the combination of pulse parameters delivered by the ablation catheter can enhance myocardial ablation efficacy while minimizing vascular injury. Additionally, our findings indicate that increased fat thickness and smaller vascular radius reduce the penetration efficiency of the ablation electric field (Wang et al., 2024b; Gasperetti et al., 2023). The study also shows that fat similarly impedes thermal conduction. In patients with complex cardiac conditions, such as those with coronary artery disease and thick epicardial fat, increasing the ablation catheter pulse voltage may help achieve effective lesion formation (Cho et al., 2018).

Since the ablation catheter is positioned directly within the blood vessel, its metal electrodes make direct contact with the vascular wall during energy delivery, resulting in a rapid temperature rise in the ablation zone. This temperature increase is significantly greater than that observed in prior epicardial ablation simulations (González-Suárez et al., 2022b; Jiang et al., 2024). Therefore, temperature dynamics in the ablation zone should be carefully considered in vascular simulation studies and animal experiments. Adequate intervals between successive ablation pulses should be maintained to allow thermal recovery within the ablation zone.

### 4.2 Other related recommendations

Research has shown that arterial spasms can occur during the use of PFA for arrhythmia treatment. This phenomenon may involve several mechanisms, including high voltages that alter the membrane potential of vascular smooth muscle cells and electric fields that stimulate the perivascular sympathetic nerves, thereby promoting vasoconstriction (Malyshev et al., 2024; Reddy et al., 2022). In our study, we simulated placement of the ablation catheter inside the vessel, which may place it closer to the coronary artery. Therefore, this may further increase the likelihood of arterial spasms during ablation. Our investigation also found that arterial spasms can occur with other ablation methods, such as cryoablation and radiofrequency ablation, used for arrhythmia treatment. This phenomenon does not cause permanent vascular damage, and nitroglycerin can effectively relieve the symptoms (Keelani et al., 2025; Hachisuka et al., 2022; Ladejobi et al., 2022; Yajima et al., 2018). As this is a prospective study, we recommend a stepwise energy escalation protocol in animal experiments and future clinical applications. Electrocardiogram data should be closely monitored to reduce the risk of complications such as arterial spasm.

Our research team conducted animal experiments using ablation catheters on the superior vena cava, inferior vena cava, and anterior interventricular vein. The ablation process was smooth, and the results showed that the pulsed energy effectively penetrated blood vessels and fat to induce electric field damage to the myocardium. No significant damage was observed in nearby small arteries or surrounding organs, further supporting the efficacy and safety of this technology (Xuan et al., 2025). However, the results of this animal study have certain limitations. We acknowledge that differences exist in tissue parameters and structural characteristics between animals and humans. Nevertheless, this animal study demonstrates that endovascular catheter ablation has potential clinical applications.

The intravascular catheter ablation technique proposed in this study may be used in the future to treat deep myocardial lesions that are difficult to reach with traditional ablation methods, such as intraventricular tachycardia circuits within the ventricular wall or focal arrhythmias in the interventricular septum. Intravascular ablation can deliver the catheter directly to deep myocardial layers through vascular pathways such as the coronary sinus or septal perforating branches, enabling more precise pulsed electric field application. Additionally, arrhythmias that originate from vascular regions, such as ventricular arrhythmias from the summit region and atrial arrhythmias involving the Marshall vein, remain challenging to treat with conventional methods like cryoablation or radiofrequency ablation, often resulting in suboptimal efficacy (Muser and Santangeli, 2020; Briceño et al., 2019; Tam et al., 2022; Müller et al., 2023). This study leverages the tissue selectivity of PFA, and the proposed technique may help improve treatment outcomes for these arrhythmias.

It is worth noting that the electrical conductivity values of biological tissues used in this simulation may differ slightly from those reported in other studies. This variation is mainly due to differences in study populations and research methods (Meckes et al., 2022; González-Suárez et al., 2022b; González-Suárez and Berjano, 2015; Pérez and González-Suárez, 2023). The extent of damage in the ablation zone is affected by factors such as tissue electrical conductivity, which influences the electric field distribution, and perfusion rate, which can promote temperature elevation in the ablation area. Notably, the relative trend in tissue conductivities (e.g., myocardial conductivity being higher than fat conductivity) remains consistent despite individual differences. In addition, studies have shown that fat and scaffolds in the ablation region can alter the electric field distribution. Scaffolds can also enhance temperature rise in the ablation zone (González-Suárez et al., 2022b; Pérez and González-Suárez, 2023). Therefore, further animal studies may be needed to verify safety in relevant patient populations. To prevent thermal damage to blood vessels, appropriate pulse parameters are essential for controlling temperature in the ablation zone.

### 4.3 Limitations

In this simulation, we used the computer to establish a three-dimensional computational model to simplify the model of the targeted ablation area, at the same time, the ablation catheter was dimensionally reduced, and the finite element was used to analyze and compare the temperature distribution of the ablation area and the electric field distribution of the ablation catheter in the intravascular ablation under different parameters. However, this study only collected and analyzed data from the cross-section of the ablation catheter, which may limit the analysis of certain damage in the ablation area. We focused on the width and depth of the effective ablation area, as these are key factors of interest for researchers in clinical applications. However, in practice, the ablation zone is a three-dimensional volume. Because our study mainly focuses on whether catheter ablation damages blood vessels, we consider cross-sectional measurement data more suitable for description and analysis. Our simulation study is the first to simulate endovascular ablation with an ablation catheter. We analyzed multiple data controls, including ablation voltage, fat thickness, and vessel (cardiac vascular) radius, generating rich ablation data that provides valuable insights for subsequent animal studies.

Finally, although our team conducted animal experiments to assess the safety and feasibility of this new ablation method, we acknowledge that the number of experiments was limited. It should also be noted that structural differences may exist between animals and humans. Our study is based on the physics of the model and simulation analysis, we encourage future researchers to verify our results through animal experiments based on our simulation calculations.

## 5 Conclusion

Some studies have shown that the PFA technique has unique advantages in the treatment of arrhythmias (high ablation precision, non-thermal effect ablation, and short recovery period time, etc.), and researchers have achieved satisfactory results by isolating the pulmonary veins for the treatment of atrial fibrillation, and also epicardial pulsed-field ablation for the treatment of arrhythmias is undergoing further study, but ablation from the cardiac vasculature to achieve some of the symptoms of arrhythmias has not yet been by the study However, the study of partial arrhythmia symptoms from cardiac vascular ablation has not yet been studied. We established a three-dimensional computational simulation model by computer and calculated the electric field distribution and temperature distribution in the ablation area during catheter ablation in the vasculature by using finite element analysis, which provides a new idea for the treatment of arrhythmia originating from the depth of the myocardium. Through the three-dimensional model calculation, it is known that: 1. Cardiac vascular ablation has certain feasibility in the treatment of arrhythmia; 2. It is necessary to carefully regulate the voltage of the ablation electrode in the ablation, to avoid damage to the blood vessels; 3. Since the ablation catheter is located in the inner part of the blood vessels, the temperature of the ablation area is warmed up obviously, and it is recommended to reasonably configure the ablation pulse interval.

## Data Availability

The original contributions presented in the study are included in the article/supplementary material, further inquiries can be directed to the corresponding authors.
